# Characterization of a Novel Plasmid, pMAH135, from *Mycobacterium avium* Subsp. *hominissuis*

**DOI:** 10.1371/journal.pone.0117797

**Published:** 2015-02-11

**Authors:** Kei-ichi Uchiya, Hiroyasu Takahashi, Taku Nakagawa, Tetsuya Yagi, Makoto Moriyama, Takayuki Inagaki, Kazuya Ichikawa, Toshiaki Nikai, Kenji Ogawa

**Affiliations:** 1 Department of Microbiology, Faculty of Pharmacy, Meijo University, Nagoya, Japan; 2 Department of Pharmacy, Kainan Hospital Aichi Prefectural Welfare Federation of Agricultural Cooperatives, Yatomi, Japan; 3 Department of Clinical Research, National Hospital Organization, Higashi Nagoya National Hospital, Nagoya, Japan; 4 Department of Pulmonary Medicine, National Hospital Organization, Higashi Nagoya National Hospital, Nagoya, Japan; 5 Department of Infectious Diseases, Center of National University Hospital for Infection Control, Nagoya University Hospital, Nagoya, Japan; 6 Department of Pharmacy, National Hospital Organization, Toyohashi Medical Center, Toyohashi, Japan; 7 Department of Pharmacy, Nagoya University Hospital, Nagoya, Japan; Cornell University, UNITED STATES

## Abstract

*Mycobacterium avium* complex (MAC) causes mainly two types of disease. The first is disseminated disease in immunocompromised hosts, such as individuals infected by human immunodeficiency virus (HIV). The second is pulmonary disease in individuals without systemic immunosuppression, and the incidence of this type is increasing worldwide. *M. avium* subsp. *hominissuis*, a component of MAC, causes infection in pigs as well as in humans. Many aspects of the different modes of *M. avium* infection and its host specificity remain unclear. Here, we report the characteristics and complete sequence of a novel plasmid, designated pMAH135, derived from *M. avium* strain TH135 in an HIV-negative patient with pulmonary MAC disease. The pMAH135 plasmid consists of 194,711 nucleotides with an average G + C content of 66.5% and encodes 164 coding sequences (CDSs). This plasmid was unique in terms of its homology to other mycobacterial plasmids. Interestingly, it contains CDSs with sequence homology to mycobactin biosynthesis proteins and type VII secretion system-related proteins, which are involved in the pathogenicity of mycobacteria. It also contains putative conserved domains of the multidrug efflux transporter. Screening of isolates from humans and pigs for genes located on pMAH135 revealed that the detection rate of these genes was higher in clinical isolates from pulmonary MAC disease patients than in those from HIV-positive patients, whereas the genes were almost entirely absent in isolates from pigs. Moreover, variable number tandem repeats typing analysis showed that isolates carrying pMAH135 genes are grouped in a specific cluster. Collectively, the pMAH135 plasmid contains genes associated with *M. avium*’s pathogenicity and resistance to antimicrobial agents. The results of this study suggest that pMAH135 influence not only the pathological manifestations of MAC disease, but also the host specificity of MAC infection.

## Introduction

Many species of nontuberculous mycobacteria (NTM) are environmental inhabitants distributed in natural water systems and soil, as well as in residential dust, water distribution systems, and bathrooms [1,2]. NTM infection is thought to be caused by NTM in residential soil or bathrooms [3,4], and the prevalence of NTM lung disease is increasing worldwide. More than 20 NTM species can cause infection in humans, but causative NTM species vary by country. In Japan, *Mycobacterium avium* complex (MAC) accounts for approximately 80% of the causative species, followed by *M*. *kansasii* and *M*. *abscessus* [5].

MAC comprises two closely related species, *M*. *intracellulare* and *M*. *avium*. The latter consists of four subspecies (*M*. *avium* subsp. *avium*, *M*. *avium* subsp. *silvaticum*, *M*. *avium* subsp. *hominissuis*, and *M*. *avium* subsp. *paratuberculosis*) that each infect specific hosts. Among these subspecies, *M*. *avium* subsp. *hominissuis*, which has been mainly isolated from environmental sources, humans, and pigs, is thought to be the causative agent of MAC disease in humans [6]. Analyses carried out in Europe using molecular genotyping methods showed high genetic similarity between the *M*. *avium* strains isolated from humans and pigs [7–10]. These results indicate the possibility that there is a common source of *M*. *avium* infection in pigs and in humans, as well as for pig–human zoonotic infection.

*M*. *avium* is an opportunistic pathogen that causes generalized disseminated disease in immunocompromised patients, such as human immunodeficiency virus (HIV)-positive patients. In contrast to HIV-associated disseminated disease, pulmonary disease in immunocompetent individuals is caused by *M*. *intracellulare* or *M*. *avium*, and the prevalence of each varies by country. In Japan, the incidence of pulmonary disease caused by *M*. *avium* is about 7 times higher than that caused by *M*. *intracellulare* [5]. A previous study showed that the *M*. *avium* strains that cause pulmonary and disseminated disease possess genetically distinct features which may influence the pathological manifestations of MAC disease [11]. Pulmonary MAC disease is divided into two major disease types, the nodular bronchiectatic type and the fibrocavitary type, with the former being more prevalent [12]. In recent years, a form of pulmonary MAC disease characterized by lesions in the lingular segments and middle lobe has been increasing in many countries in middle-aged to elderly females with no underlying disease [13]. The high prevalence of pulmonary MAC disease in this female population and the variable clinical manifestations may involve bacterial factors in addition to host-related risk factors [14]. Although host-related risk factors have been investigated, no definitive evidence has been found and little is yet known about bacterial molecular factors.

Many bacterial species possess a plasmid, a genetic unit capable of replicating independently of the chromosome. Plasmids have been shown to contain important genes that determine bacterial virulence and resistance to antimicrobial agents including antibiotics. With regard to mycobacterial plasmids, previous studies isolated pAL5000 [15] and pJAZ38 [16] from *M*. *fortuitum* and pMSC262 [17] from *M*. *scrofulaceum*. In addition, two types of plasmids were isolated from *M*. *avium*, pVT2 [18] and pLR7 [19], the latter of which was from a strain isolated from AIDS patients and has no homology to pVT2. Because of their relatively small size of 4.8–16 kb and the absence of virulence genes, the significance of these plasmids is currently unknown. Stinear et al. isolated pMUM001, a 174-kb giant plasmid containing virulence genes, from *M*. *ulcerans* [20]. pMUM001 contains genes that are involved in the synthesis of a macrolide toxin, called mycolactone, which exhibits cytotoxic, analgesic, and immunosuppressive activities. Furthermore, plasmids isolated from *M*. *marinum* and *M*. *abscessus* contain mercury resistance genes [21,22].

In this study, we determined the complete sequence of pMAH135 from *M*. *avium* strain TH135 isolated from a patient with pulmonary MAC disease. We then investigated the characteristics of pMAH135 by bioinformatics analysis and screening of *M*. *avium* isolates for pMAH135 genes, followed by variable number tandem repeats (VNTR) typing analysis.

## Materials and Methods

### Bacterial strains

*M*. *avium* subsp. *hominissuis* strain TH135 isolated from the sputum of an HIV-negative patient with pulmonary MAC disease was used for genome analysis. *M*. *avium* isolates used for screening of pMAH135 genes comprised 35 clinical isolates including strain TH135 collected from the sputa of HIV-negative patients with pulmonary MAC disease at Higashi Nagoya National Hospital of the National Hospital Organization in Japan, from 2004 to 2008; 29 clinical isolates from blood of HIV-positive patients with MAC disease provided by the National Center for Global Health and Medicine, formerly the International Medical Center of Japan; and 23 isolates from pig tissue from the Meat Hygiene Inspection Center of Okinawa Prefecture, provided by Ryukyu University. *M*. *avium* strain 104, derived from an AIDS patient, was used as standard strain [23].

### Identification of *M*. *avium* subsp. *hominissuis*, growth condition, and DNA isolation

The subspecies of *M*. *avium* isolates was identified as *M*. *avium* subsp. *hominissuis* by sequence analysis of the 3´ fragment of the *hsp65* gene [24]. The organism was grown in Middlebrook 7H9 liquid medium supplemented with 10% oleic acid/albumin/dextrose/catalase enrichment (Difco Laboratories, Detroit, MI) at 37°C. DNA was extracted with a Qiagen kit (Qiagen Inc., Valencia, CA) according to the manufacturer’s instructions using 50,000 U/mL lipase, 5 mg/mL lysozyme, and 5 mg/mL proteinase K (Sigma-Aldrich, St. Louis, MO).

### DNA sequencing, annotation, and bioinformatics analysis

The DNA sequence of the isolate from *M*. *avium* strain TH135 was determined as described below, and the result showed the presence of a circular plasmid independent of the genome DNA (GenBank accession no. AP012555). DNA sequencing was performed by combining the technology of two genome sequencers: 454 GS FLX (Roche, Mannheim, Germany); and Hiseq 2000 (Illumina, CA). DNA was sequenced using the Hiseq with a 101-bp paired-end library (80,119,704 reads, 1,600-fold genome coverage) and the FLX with 8-kb paired-end reads (295,431 reads, 14-fold genome coverage), and total sequence reads were assembled using GS De Novo Assembler (version 2.8). Gap sequences in the scaffolds were filled by polymerase chain reaction (PCR) amplification followed by Sanger sequencing. The DNA sequence was automatically annotated using the Microbial Genome Annotation Pipeline [25] and corrected manually using *in silico* Molecular Cloning Genomics Edition (IMCGE) software [26].

Protein function was assigned based on a BLASTP similarity search against the NCBI ‘nr’ (non-redundant protein) database. Transfer RNA (tRNA) and ribosomal RNA (rRNA) were predicted using tRNAscan-SE 1.23 and RNAmmer 1.2, respectively. Insertion sequence (IS) elements were identified using IS-Finder [27], and data management and visualization of plasmid features were performed using the IMCGE software.

### Plasmid analysis

Plasmid DNA analysis was carried out by S1-pulsed-field gel electrophoresis (PFGE) [28]. Total DNA in agarose gel plugs was prepared as described previously [29] with the following modifications. Each isolate was grown in 30 mL 7H9 liquid medium supplemented with 10% oleic acid/albumin/dextrose/catalase enrichment at 37°C. When cell suspensions reached an optical density of 0.2–0.3 at 600 nm, cells were harvested by centrifugation, washed in modified spheroplasting buffer [29], and resuspended in this buffer. The suspension was mixed with an equal volume of 1.5% low-melting-point agarose and poured into plug molds (Bio-Rad Laboratories, Hercules, CA). After lysozyme and proteinase K digestion, plugs were washed in Tris-EDTA buffer (pH 8.0) and stored at 4°C until used for PFGE analysis. Slices 3–5 mm thick were cut from the plugs, washed in 10 mM Tris-HCl buffer (pH 8.0), and treated with 10 U S1 nuclease (Takara Bio, Shiga, Japan) for 10 min at 37°C. S1 nuclease-digested plug sections were loaded into 1% agarose gel in 0.5% TBE buffer (Bio-Rad Laboratories), and electrophoresis was carried out in a CHEF-DR III system (Bio-Rad Laboratories) at 14°C and 6 V/cm^2^ for 24 h with a switch time of 1.6–21.3 s. The Lambda Ladder PFG Marker (New England Biolabs, Ipswich, MA) was used as a molecular size standard. After electrophoresis, DNA fragments were transferred to positively charged nylon membranes (GE Healthcare, Buckinghamshire, UK) and fixed to the membrane by UV cross-linking (150 mJ/cm^2^). Southern hybridization analysis including probe labeling was performed using the DIG High Prime DNA Labeling and Detection Starter Kit II (Roche) according to the manufacturer’s instructions. A pMAH135-specific probe was prepared by PCR with the primers for MAH_p01 (5′-AAA GAC GCA TTC CAC GGT AG-3′ and 5′-GGG GAG GTT TTA GGG AGA AA-3′) and DNA from *M*. *avium* strain TH135.

### Molecular typing

VNTR typing analysis using *M*. *avium* tandem repeats (MATR) was carried out as previously described [30]. After the number of base pairs in the target VNTR loci was estimated by agarose gel electrophoresis in relation to molecular weight markers, the number of repetitions of various VNTR loci in each strain was determined and regarded as an allele profile. The Manhattan distance was determined on the basis of each obtained allele profile, and a dendrogram was estimated by the Fitch-Margoliash method prepared in FigTree (version 1.3.1).

### PCR and sequence analyses

*M*. *avium* isolates were cultured in 5 mL 7H9 liquid medium supplemented with 10% oleic acid/albumin/dextrose/catalase enrichment at 37°C for 1–2 weeks. The culture was centrifuged and DNA was extracted using InstaGene Matrix (Bio-Rad Laboratories) according to the manufacturer’s instructions. PCR was performed essentially as described previously [11]. The PCR primers used in this study are shown in S1 Table. The resulting PCR products were purified using a GenElute PCR DNA purification kit (Sigma-Aldrich), and direct sequencing analysis was performed using the same primers as those used for PCR. The resulting nucleotide sequences were compared with the sequence data of pMAH135. The suitability of the present DNA samples for screening clinical isolates by PCR was determined by amplification of the *hsp65* gene—the gene used to identify subspecies of *M*. *avium* isolates.

### Statistical analysis

Data for the detection rate of pMAH135 genes in *M*. *avium* isolates were analyzed statistically using Fisher’s exact test. All statistical analysis was performed using the GraphPad Prism version 5.0 (GraphPad Software, San Diego, CA). *P* values <0.05 were considered significant.

### Nucleotide sequence accession number

The complete sequence of pMAH135 was deposited in DDBJ/EMBL/GenBank under accession no. AP012556.

### Ethics Statement

This study was approved by the Ethics Review Committee for Human Research of the Higashi Nagoya National Hospital, and written informed consent was obtained from all patients.

## Results and Discussion

### Identification of pMAH135

**Fig 1:**
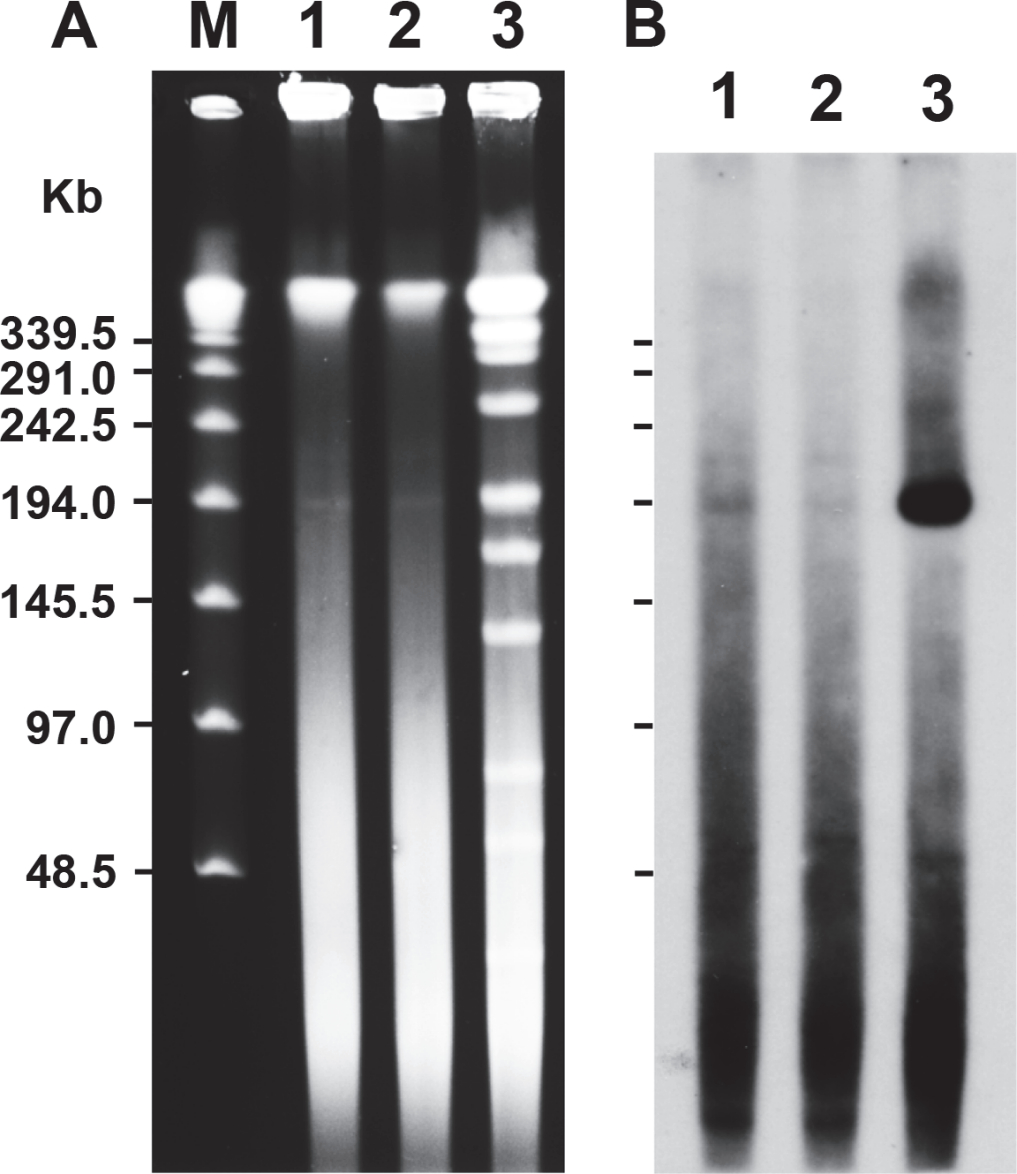
Demonstration of the pMAH135 plasmid. Pulsed-field gel electrophoresis (PFGE) of total DNA of *Mycobacterium avium* strain TH135 (A) and Southern hybridization with a probe derived from MAH_p01 specific to pMAH135 (B). Lane 1, S1 nuclease-digested DNA; lane 2, undigested DNA; lane 3, *Ase*I-digested DNA; lane M, the lambda ladder PFG marker.

Genomic sequencing of *M*. *avium* strain TH135 isolated from a patient with pulmonary MAC disease demonstrated the presence of a circular plasmid, designated pMAH135. Furthermore, to confirm the presence of pMAH135, we carried out PFGE analysis by treatment with S1 nuclease, which converts supercoiled plasmids into full-length linear DNA molecules [28]. As shown in Fig. 1A, a band was observed close to 194 kb. The band closely matched the size of pMAH135 as determined by sequence analysis (Table 1). To verify that this band was indeed pMAH135, we performed Southern hybridization analysis. A 662-bp fragment derived from MAH_p01 that was specific to the plasmid and not found on the chromosome of strain TH135 was used as the probe. The results confirmed that the band was pMAH135 (Fig. 1B).

**Table 1:** General features of pMAH135.

Property	pMAH135
Plasmid size, bp	194,711
G + C content, %	66.5
Protein coding, %	84.5
ORFs	164
Average gene length, bp	1003
tRNAs	1
rRNA operons (16S-23S-5S)	0
Prophage elements, no.	1
IS, total no. of copies	6

CDS, coding sequence; tRNA, transfer RNA

rRNA, ribosomal RNA; IS, insertion sequence.

However, because circular plasmids move much more slowly than their corresponding linear forms in PFGE, the observation of a band at the same position in a PFGE analysis without S1 nuclease treatment suggested that pMAH135 may be a linear plasmid. PFGE analysis was therefore performed using an *Asel* restriction enzyme. *Asel* cleaves pMAH135 at a single position 8,095 bp downstream of the *repA* (MAH_p01) initiation codon. The presence of a band was confirmed at a similar size position, as shown in Fig. 1B. Because in a linear plasmid, no bands should be observed at this position, this finding indicated that pMAH135 is circular. This is strongly supported by the fact that reads obtained from DNA sequencing analysis using two genome sequencers revealed a complete coverage of the pMAH135 sequence, including the adjoining section with start and end points after assembly (data not shown). The cause of a band of same size appearing in PFGE analysis without S1 nuclease treatment is unknown; however, one possible explanation is that pMAH135 was cleaved physically. There has in fact been a case of a band corresponding to the plasmid size of pMUM001 (174,155 bp) from *M*. *ulcerans* being confirmed in a PFGE analysis without S1 nuclease treatment, even though it is a circular plasmid [20].

### General features of pMAH135

**Fig 2:**
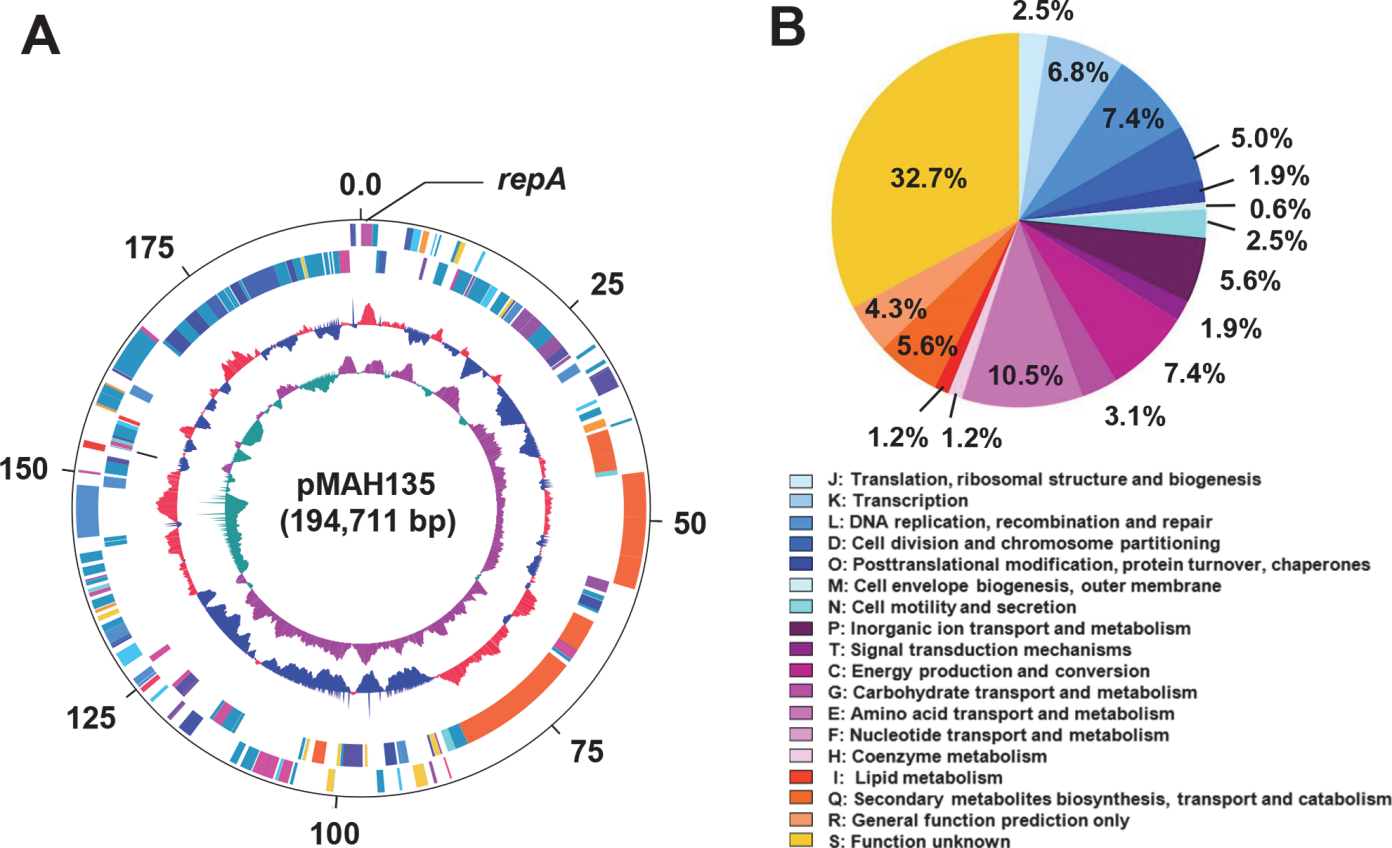
Circular representation of the pMAH135 plasmid and cluster of orthologous groups (COG) functional classification of pMAH135 genes. (A) Circular representation of pMAH135 from *M*. *avium* strain TH135. Scale is shown in kilobase pairs by the black outer circle. From the outside to the inside, the outer two circles show forward- and reverse-strand coding sequences (CDSs), respectively. The third circle shows tRNA genes. The fourth circle shows the percentage of G + C content in relation to the mean G + C content of the chromosome. The fifth circle shows the GC skew ((G—C) / (G + C)). The color of each CDS was assigned according to the COG functional classification system [35]. (B) Classification of genes located on pMAH135 based on COG functional categories. Each colored segment indicates the relative contribution of each functional category as a percentage of total COGs. The color of each COG family is shown in the figure.

The general features of pMAH135 are presented in Table 1. This plasmid was composed of 194,711 base pairs with an average G + C content of 66.5%, 164 predicted coding sequences (CDSs), 1 tRNA gene, and 6 IS elements. This G + C content was characteristically low compared with that of the chromosome (69.3%), suggesting that the plasmid had been transformed into the cell at some point during the evolutionary process. pMAH135 was unique in terms of homology to other mycobacterial plasmids. The replication origin of pMAH135 was deduced on the basis of the presence of predicted product of the *repA* gene (Fig. 2A) [31], which shares 56% amino acid identity with RepA encoded by the *M*. *avium* plasmid pLR7. In many cases, the replication origin contains direct repeat sequences, termed iterons, which are the binding sites for the Rep proteins [31]. Analysis of the replication region of pMAH135 revealed the presence of a single pair of 14-bp iterons (5′-GGC GGA TAT CCG CC-3′) in the region 211–252 bp upstream of the *repA* initiation codon. The present study suggests that pMAH135 is a plasmid-like element, more likely, an autonomously replicating plasmid. However, because the present study does not address whether this plasmid is capable of autonomous replication, further assays are necessary to determine the presence of a functional replicon.

The region approximately 1 kb downstream of *repA* (MAH_p01) contains homologues of *parA* and *parB* (S2 Table), which are partitioning genes required for plasmid segregation during cell division [32]. ParA is an ATPase stimulated by ParB, which is a DNA-binding protein. Interestingly, the plasmid contained two CDSs, MAH_p04 and MAH_p05, which encode a ParA homologue and have 92% and 90% amino acid identity with ParA from *M*. *parascrofulaceum* and *M*. *tusciae*, respectively. On the other hand, MAH_p06 encoding the ParB homologue has 78% amino acid identity with ParB from *M*. *parascrofulaceum*. Furthermore, MAH_p07 and MAH_p08 in this region encode proteins with significant similarity to plasmid stability proteins in *M*. *parascrofulaceum*. These components, including ParA and ParB, are required for plasmid stability.

IS elements are thought to be one of the major players in prokaryote genome plasticity [33]. A greater number of IS elements indicates that the genome has undergone further structural variation during strain evolution. We previously showed that five IS*Mav6* genes with 60 point mutations relative to the subspecies differentiation marker IS*901*, which is on the genomes of different subspecies (*M*. *avium* subsp. *avium* and *M*. *avium* subsp. *silvaticum*), are coded by the strain TH135 chromosome [34]. Two of the six IS elements on pMAH135 (MAH_p35 and MAH_p136) were IS*Mav6* genes, demonstrating that strain TH135 harbors seven characteristic IS*Mav6* genes.

BLAST analysis revealed that 47.8% of the protein CDSs in pMAH135 showed the highest homology to proteins coded by the *M*. *parascrofulaceum* chromosome, and 5.5% and 4.9% of the protein CDSs in pMAH135 were homologous to proteins in *M*. sp. MOTT36Y and *M*. *indicus pranii*, respectively (S2 Table). Furthermore, when we classified CDSs on pMAH135 according to the cluster of orthologous groups category [35], those as large as 32.7% of the CDSs were classified as category S (function unknown), followed by 10.5% as category E (amino acid transport and metabolism), and 7.4% each as category L (recombination and repair) and category C (energy production and conversion) (Fig. 2B). On the other hand, none of the CDSs belonged to category F (nucleotide transport and metabolism).

Of the pMAH135 CDSs, attention must be paid to those encoding proteins involved in mycobactin biosynthesis and the type VII secretion system, both of which are important to the virulence of mycobacteria as well as to proteins with putative conserved domains of the multidrug efflux transporter (Table 2). We review these factors in detail below.

**Table 2:** Comparison of virulence- and drug efflux-related proteins in the *Mycobacterium tuberculosis* H37Rv genome, *M*. *avium* TH135 genome, and pMAH135.

	Related gene	*M*. *tuberculosis* H37Rv	*M*. *avium* TH135	pMAH135
		Locus tag	Locus tag	Locus tag
Mycobactin	*mbtH*	Rv2377c	MAH_1634 (70.6%) ^a^ MAH_2755 (78.3%)	
	*mbtG*	Rv2378c	MAH_1633 (88.7%)	
	*mbtF*	Rv2379c	MAH_1632 (60.0%)	MAH_p48 (41.7% ^b^/43.6% ^c^)
	*mbtE*	Rv2380c	MAH_1631 (65.3%)	MAH_p47 (44.9%/44.0%)
	*mbtD*	Rv2381c	MAH_1630 (46.9%)	MAH_p43 (34.9%/35.3%)
	*mbtC*	Rv2382c	MAH_1629 (71.7%)	MAH_p44 (48.6%/50.8%)
	*mbtB*	Rv2383c	MAH_1627 (71.6%)	MAH_p49 (51.3%/51.1%)
	*mbtA*	Rv2384	MAH_1626 (78.5%)	
	*mbtJ*	Rv2385	MAH_1590 (68.7%)	
	*mbtI*	Rv2386c	MAH_1584 (73.8%)	
	*mbtL*	Rv1344	MAH_2416 (76.5%)	
	*mbtM / fadD33*	Rv1345	MAH_2415 (70.2%)	
	*mbtN / fadE14*	Rv1346	MAH_2414 (82.2%)	
	*mbtK*	Rv1347c	MAH_2413 (74.6%)	
	*irtA*	Rv1348	MAH_1369 (79.8%)	
	*irtB*	Rv1349	MAH_1370 (77.3%)	
ESX-5	*eccA5*	Rv1798	MAH_2374 (89.3%)	MAH_p142 (71.6%/71.0%)
	*eccE5*	Rv1797	MAH_2373 (65.2%)	MAH_p143 (33.3%/36.4%)
	*mycP5*	Rv1796	MAH_2372 (71.0%)	MAH_p144 (58.3%/65.5%)
	*eccD5*	Rv1795	MAH_2371 (79.3%)	MAH_p145 (49.4%/51.6%)
	*espG5*	Rv1794	MAH_2370 (93.7%)	MAH_p147 (43.8%/43.4%)
	*esxN*	Rv1793	MAH_2369 (93.6%)	MAH_p148 (91.5%/95.7%) MAH_p51 (90.4%/92.6%)
	*esxP*	Rv2347c	MAH_2368 (87.8%)	MAH_p149 (91.0%/94.9%) MAH_p52 (81.6%/88.8%)
	*eccC5*	Rv1783	MAH_2355 (90.0%)	MAH_p152 (54.3%/54.7%)
	*eccB5*	Rv1782	MAH_2354 (82.6%)	MAH_p153 (57.5%/58.1%)
	*PPE41*	Rv2430c		
	*PPE25*	Rv1787	MAH_2564 (43.9%)	
	*PE18*	Rv1788	MAH_2367 (89.7%)	MAH_p54 (72.4%/71.3%)
	*PPE26*	Rv1789	MAH_2361 (55.5%)	
	*PPE27*	Rv1790		
	*PE19*	Rv1791		
PPE or PE	*PPE19*	Rv1361c	MAH_1624 (47.9%)	MAH_p53 (42.3%/50.3%)MAH_p150 (42.2%/37.7%)
	*PPE32*	Rv1808	MAH_2379 (55.6%)	MAH_p70 (46.4%/71.6%)
	*PPE3*	Rv0280	MAH_2622 (66.5%)	MAH_p91 (49.2%42.3%)
	*PE31*	Rv3477	MAH_2375 (67.8%)	MAH_p151 (47.4%/44.4%)
Drug efflux protein	*emrB*	Rv0783c	MAH_0637 (66.5%)	MAH_p59 (54.4%/57.4%)
MATE efflux protein				MAH_p85

^a^Amino acid sequence identity of virulence- and drug efflux-related proteins in *M*. *aviu*m TH135 in relation to thecorresponding proteins in *M*. *tuberculosis* H37Rv

^b^Amino acid sequence identity of virulence- and drug efflux-related proteins in pMAH135 in relation to the corresponding proteins in *M*. *tuberculosis* H37Rv

^c^Amino acid sequence identity of virulence- and drug efflux-related proteins in pMAH135 in relation to the corresponding proteins in *M*. *avium* TH135

Empty cells indicate no homologous virulence- and drug efflux-related proteins

### ESX system

Pathogenic mycobacteria carry the type VII secretion system membrane complex, termed the ESX system, to transport virulence factors across their cell envelope, and to date, five types of ESX systems, ESX-1 to ESX-5, have been reported [36,37]. ESX-1 is responsible for secreting 6-kDa early secreted antigenic target (ESAT-6), which can disturb the activation of macrophages, induce apoptosis, and subvert host immunity, and its protein partner the 10-kDa culture filtrate protein-10, thereby contributing to the virulence of pathogenic mycobacteria [36,38,39]. On the other hand, ESX-3 plays a role in iron transport and is thus essential for bacterial viability [40,41]. ESX-5, which is the most-recently evolved type VII secretion system, mediates the secretion of ESAT-6-like protein EsxN and mycobacteria-specific proteins with conserved N-terminal domains containing prolyl-glutamic acid (PE) or prolyl-prolyl glutamic acid (PPE) motifs [42–44]. The *M*. *marinum* ESX-5 system is involved in inducing cell death of infected macrophages and modulating the immune response [36,45]. However, unlike ESX-1, ESX-5 does not affect the escape of mycobacteria from phagolysosomes into the cytosol of infected macrophages [36,46]. Houben et al. proposed the ESX secretion system model after analyzing ESX-related proteins encoded by the *esx-5* locus in *M*. *tuberculosis* [44]. ESX core components (Ecc)B–E are embedded in the cytosolic membrane where they are assembled into a large membrane complex. In particular, EccC and EccD are essential for the secretion of various substrates. EccC, a member of the FtsK/SpoIIIE family of ATPases, is likely involved in energizing the translocation of substrates.

Comparative analysis revealed that pMAH135 contained CDSs with a 33.3–91.5% sequence identity to ESX-related proteins encoded by the *esx-5* locus in the *M*. *tuberculosis* H37Rv genome (GenBank accession no. NC_000962) (Table 2). These CDSs also share 36.4–95.7% sequence identity to ESX-5-related proteins encoded by the TH135 chromosome. Interestingly, MAH_p51 and MAH_p148 were highly homologous to EsxN in *M*. *tuberculosis* H37Rv, and MAH_p52 and MAH_p149 were highly homologous to EsxP in *M*. *tuberculosis* H37Rv, even though the functions of EsxN and EsxP, which are both ESAT-6 homologues, are unknown. Strain TH135 possesses three genes—one on the chromosome and two on the plasmid—that are highly homologous to the *esxN* or *esxP* genes in *M*. *tuberculosis* H37Rv. As described above, because ESX-5 is involved in the virulence of pathogenic mycobacteria, it is likely that the ESX-5-related proteins encoded by pMAH135, together with those in the chromosome, contribute to the pathogenicity of strain TH135.

Mycobacteria carry many genes encoding PPE and PE proteins with unknown function. In particular, several CDSs (MAH_p53, MAH_p150, MAH_p70, MAH_p91, and MAH_p151) showing a 42.2–49.2% sequence identity to PPE19, PPE32, PPE3, and PE31 in *M*. *tuberculosis* H37Rv were found on pMAH135 (Table 2). It will be interesting to determine if any of these factors are involved in pathogenesis.

### Mycobactin

Iron is an essential nutrient for almost all organisms. Like many bacterial pathogens, mycobacteria synthesize siderophores to capture iron, which is present in limited concentrations in living hosts [47]. Pathogenic mycobacteria, including *M*. *tuberculosis* and *M*. *avium*, utilize two forms of siderophores with a 2-hydroxyphenyloxazoline moiety; these are termed carboxymycobactin and mycobactin and differ according to the length of their alkyl substitution [48]. During cell culture, carboxymycobactin (the more polar form) is released into the medium, whereas mycobactin (the less polar form) remains cell associated. The loci involved in iron acquisition via siderophores comprise the siderophores biosynthesis genes *mbtA-N* and *irtAB* that encode iron-regulated transporters [47,49]. De Voss et al. reported that an *M*. *tuberculosis* mutant lacking the *mbtB* gene, which encodes a non-ribosomal peptide synthetase, interrupted the biosynthesis of siderophores and impaired the growth of macrophages [50]. Thus, siderophores can be regarded to play a significant role in *M*. *tuberculosis* pathogenicity.

The chromosome of strain TH135 contains CDSs with a 46.9–82.2% sequence homology to proteins encoded by the *mbtA-N* and *irtAB* genes in the *M*. *tuberculosis* H37Rv genome (Table 2). In addition to these CDSs, pMAH135 contains 5 CDSs (MAH_p49, MAH_p44, MAH_p43, MAH_p47, and MAH_p48) with a 34.9–51.3% sequence identity to the MbtB to MbtF proteins of *M*. *tuberculosis* H37Rv, which are involved in the synthesis of the siderophore core (Table 2). These results suggest that the *M*. *avium* strains harboring these genes can take up iron more efficiently and may therefore be important for the onset of pathogenicity.

### Multidrug efflux transporters

Bacterial multidrug efflux transporters are classified into the following five groups according to their primary structure and mode of energy-coupling [51]: major facilitator superfamily (MFS); small multidrug resistance family; resistance nodulation cell division family; ATP-binding cassette superfamily; and multidrug and toxin extrusion (MATE) family. MATE transporters have a 12-membrane helix topology and utilize H^+^ or Na^+^ transmembrane gradients to drive substrate export [52]. Previous studies reported that fluoroquinolones such as ciprofloxacin and norfloxacin, aminoglycosides such as kanamycin and streptomycin, and even cationic dyes such as acriflavine and ethidium bromide are among the substrates removed via MATE transporters [53,54]. This demonstrates that the substrates for MATE transporters are diverse and have unrelated chemical structures.

Interestingly, protein BLAST analysis of pMAH135 CDSs identified a CDS (MAH_p85) with putative conserved domains of MATE family proteins similar to NorM from *V*. *cholerae* (S1 Fig. and S2 Table). On the other hand, the TH135 chromosome does not encode MATE family protein homologues. Amino acid sequence alignment of MAH_p85 and *V*. *cholerae* NorM showed that of the 10 amino acid residues constituting the cation-binding site in NorM [52], 4 were identical while 3 were conservative substitutions in MAH_p85 (S1 Fig.). Furthermore, MAH_p59 has a 54.4% sequence identity to the MFS transporter EmrB of *M*. *tuberculosis* H37Rv (Table 2) and a 57.4% sequence identity to MFS-like transporter MAH_0637 encoded by the TH135 chromosome. MFS proteins transport a variety of substrates including ions, simple sugars, oligosaccharides, drugs, nucleosides, amino acids, and organophosphate esters by using the electrochemical potential of the transported substrates [51,55]. It is possible that both MAH_p85 and MAH_p59 encoded by pMAH135 greatly influence the resistance of strain TH135 to antimicrobial agents including antibiotics. However, deletion mutants of these genes will be required to fully elucidate the functions of MAH_p85 and MAH_p59 and investigate their susceptibility to antimicrobial agents.

### Screening of *M*. *avium* isolates for genes located in pMAH135

To examine the prevalence of pMAH135 genes in *M*. *avium* isolates, we screened 35 clinical isolates (including strain TH135) from patients with pulmonary MAC disease, 29 clinical isolates (including strain 104) from HIV-positive patients, and 23 isolates from pigs for pMAH135 genes. As shown in Table 3, 6 genes (MAH_p47, MAH_p49, MAH_p59, MAH_p85, MAH_p143, and MAH_p148) located in pMAH135 were found in 34.3–45.7% of the clinical isolates from pulmonary MAC disease patients, compared with 10.3–20.7% of the clinical isolates from HIV-positive patients. In particular, the detection rate of MAH_p49 and MAH_p143 was significantly higher in clinical isolates from pulmonary MAC disease patients than in those from HIV-positive patients. Moreover, the rate of clinical isolates possessing all six of these CDSs was 28.6% for pulmonary MAC disease patients and 6.9% for HIV-positive patients. These results suggest that pMAH135 genes affect the pathological manifestations of MAC disease.

**Table 3:** The presence of pMAH135 genes in *M*. *avium* isolates.

Locus tag	TH^a^ (n = 35)	HIV^b^ (n = 29)	Pig^c^ (n = 23)	*P* value^d^
MAH_p47	14	6	0	0.113
MAH_p49	16	5	0	0.019
MAH_p59	12	4	0	0.083
MAH_p85	12	5	0	0.160
MAH_p143	13	3	1	0.020
MAH_p148	14	5	0	0.058
Six CDSs^e^	10	2	0	0.051

^a^ Strains from the sputa of patients with pulmonary MAC disease.

^b^ Strains from the blood of HIV-positive patients with disseminated MAC disease.

^c^ Strains from pigs.

^d^ TH-strains vs HIV-strains.

^e^ Six CDSs contained MAH_p47, MAH_p49, MAH_p59, MAH_p85, MAH_p143, and MAH_p148.

With respect to the primary route of MAC infection, disseminated disease in HIV-positive patients is most likely acquired via the gastrointestinal route. In particular, the ability of the bacteria to invade the epithelial cells lining the small intestine upon reaching the intestinal lumen is thought to be crucial to the establishment of disseminated disease [56]. On the other hand, *M*. *avium* strains that cause pulmonary disease are thought to be acquired via the respiratory route and invade via the respiratory mucosal membrane. Such strains are incorporated by phagocytosis and persist in alveolar macrophages, where they proceed to cause pulmonary disease [56]. The presence of pMAH135 genes associated with bacterial survival in host cells, namely, those genes encoding mycobactin biosynthesis proteins or ESX-5-related proteins, may be advantageous for the establishment of pulmonary disease. Comparison of the strain TH135 genome with the strain 104 genome, which was isolated from an AIDS patient, demonstrated that both strains possess genetically distinct features, and the detection rate of strain TH135-specific genes is known to be higher in clinical isolates from pulmonary disease patients than that from HIV-positive patients [11]. Taken together, these results suggest that not only strain TH135-specific genes in the chromosome, but also pMAH135 genes, have an influence on the establishment of pulmonary disease.

*M*. *avium* infects pigs as well as humans. Therefore, we examined the presence of pMAH135 genes for *M*. *avium* strains isolated from pigs. As shown in Table 3, pMAH135 genes were almost entirely absent in these isolates. As described above, *M*. *avium* infection is mainly via the respiratory or gastrointestinal routes in humans, but likely via only the gastrointestinal route in pigs. Using VNTR typing analysis, Iwamoto *et al.* showed that in Japan, the isolates from pulmonary MAC disease patients and the isolates from pigs display different genotypes [57]. This result indicates that the strains infecting pigs and humans are genetically distinct. Therefore, it can be deduced that the source of infections are different between pigs and humans, and that the possibility of zoonotic infection in Japan is low. In the present study, we find pMAH135 genes to be one of the genetic differences between the two strains. Moreover, comparison of isolates from humans and pigs from France, Finland, and Japan, using VNTR typing analysis, has indicated that the isolates from humans and pigs from France and Finland are genetically similar to the pig-derived strains from Japan, whereas the human-originated strains from Japan have a distinct genotype [9,10,57]. It thus follows that the human-derived strain from Japan is genetically distinct. Further investigation of the prevalence of pMAH135 genes among the *M*. *avium* strains from various countries may improve the understanding of their genetic diversity, and may provide a clue about the relatively high rate of *M*. *avium* infection in Japan compared with other countries.

### Relation between the VNTR genotype and distribution of pMAH135 genes

**Fig 3:**
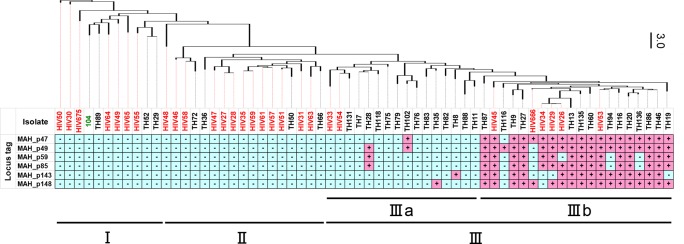
Dendrogram constructed from the results of *Mycobacterium avium* tandem repeats-variable number tandem repeat (MATR-VNTR) typing analysis and the presence of pMAH135 genes for *M*. *avium* isolates, including 63 clinical strains (TH: strains from sputa of patients with pulmonary MAC disease; HIV: strains from blood of HIV-positive patients with MAC disease) as well as strain 104 (green). The dendrogram was created from distance matrix files by Fitch-Margoliash analysis according to MATR-VNTR markers. Scale bar (right) indicates Manhattan distance. *M*. *avium* strains were classified as being clusters I–III (including strain TH135) by MATR-VNTR typing analysis. Cluster III was further classified into clusters IIIa and IIIb.

To examine the relationship between VNTR results and the distribution of pMAH135 genes, we performed VNTR typing analysis using MATR on the clinical isolates from pulmonary MAC disease patients and HIV-positive patients. Isolates from pigs scarcely contained pMAH135 genes and were therefore not included in this analysis. MATR-VNTR analysis is a rapid and highly discriminating subtyping method for *M*. *avium* [30]. As shown in Fig. 3, the *M*. *avium* isolates examined in this study were classified roughly into three clusters: cluster I, cluster II, and cluster III (including strain TH135). Cluster III was further classified into clusters IIIa and IIIb. Interestingly, pMAH135 genes were conserved in cluster III strains, especially cluster IIIb strains, albeit with a few exceptions. It is important to note that a correlation was found between the prevalence of pMAH135 genes and the MATR-VNTR genotypes. MATR-VNTR analysis could broadly distinguish between *M*. *avium* strains carrying pMAH135 genes and those that were not, and was thus found to be a useful typing method for discerning genetic variation among *M*. *avium* strains. Further genetic comparisons of strains that fall into clusters IIIb with those in clusters I, II, and IIIa may provide important information enabling prediction of pathogenesis or type of MAC disease.

### Plasmid analysis of *M*. *avium* isolates

**Fig 4:**
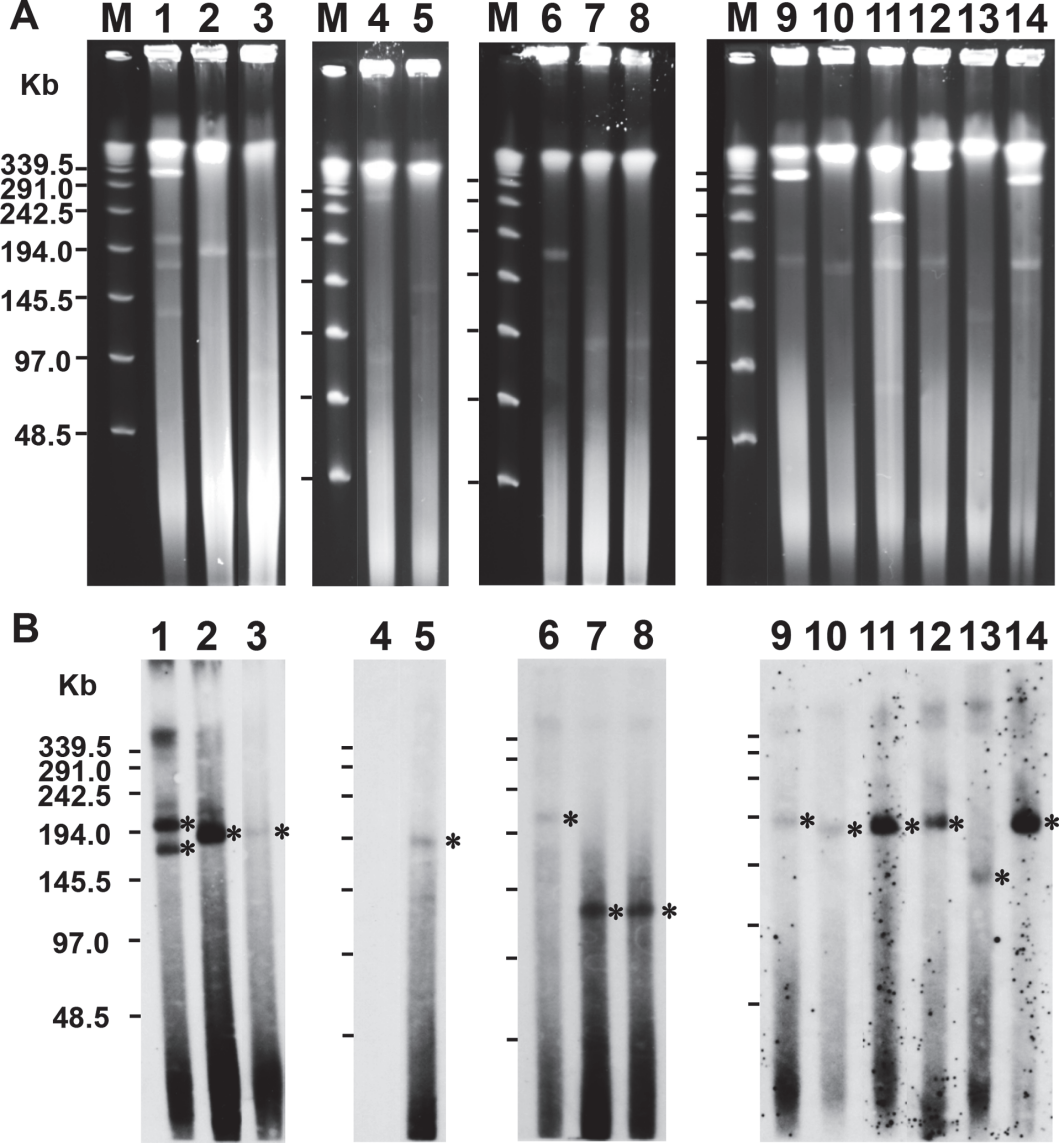
Analysis of plasmids from *M*. *avium* isolates. PFGE of S1 nuclease-digested total DNA of *M*. *avium* isolates classified in cluster IIIb as shown in Fig. 3 (A) and Southern hybridization with a probe derived from MAH_p01 (B). Asterisks show plasmid bands hybridized with the probe. Lanes 1–14 represent strains TH87, HIV45, HIV34, TH116, TH27, TH13, TH94, TH136, TH60, HIV53, TH16, TH86, TH46, and TH19. The molecular size of the lambda ladder PFG marker (lane M) is shown in the left panel.

As described above, pMAH135 genes were highly prevalent in *M*. *avium* isolates from cluster IIIb. We therefore carried out plasmid analysis of the 18 strains in this cluster and of an additional eight isolates from clusters I and II by S1-PFGE. However, precise plasmid analysis was made difficult for four of the 18 isolates from cluster IIIb (TH9, HIV666, HIV29, and HIV26) due to the low quantity of isolated DNA, despite our changing the concentration of bacteria and the concentration and treatment time of lysozyme and proteinase K (data not shown). The cause of this low quantity is unclear, but the influence of the difference in cell walls was considered a possible explanation. These four bacterial strains were excluded from the analysis as the results were deemed unreliable. Of the remaining 14 strains, the presence of the plasmid was confirmed (Fig. 4A) in 14, and eight strains (HIV45, HIV34, TH27, TH60, HIV53, TH16, TH86, and TH19) had a plasmid of almost the same size as pMAH135. Interestingly, the presence of multiple plasmids was revealed in five isolates (TH87, TH60, TH16, TH86, and TH19). As shown in Fig. 4B, eight of the 13 isolates in which the presence of MAH_p01 (used as the probe) was confirmed (HIV45, HIV34, TH27, TH60, HIV53, TH16, TH86, and TH19) had a similar size to pMAH135 and five (TH87, TH13, TH94, TH136, and TH46) contained a plasmid of a different size from pMAH135. Of the eight isolates from clusters I and II (TH89, HIV64, HIV65, TH52, HIV58, TH36, TH50, and TH66), the presence of a plasmid that differed in size from pMAH135 was revealed in four strains (HIV58, TH36, TH50, and TH66) (data not shown). The presence of a plasmid with a different size from pMAH135 is intriguing, and we plan to analyze these plasmids further and compare them with pMAH135.

## Conclusions

The pMAH135 plasmid derived from *M*. *avium* subsp. *hominissuis* strain TH135 consists of 194,711 nucleotides and encodes 164 CDSs. This plasmid was unique in terms of homology to other mycobacterial plasmids. Interestingly, pMAH135 contains genes associated with the pathogenicity of *M*. *avium* and its resistance to antimicrobial agents. Screening of *M*. *avium* isolates from humans and pigs for genes located in pMAH135 suggested that pMAH135 influence not only the pathological manifestations of MAC disease, but also the host specificity of MAC infection. In addition, *M*. *avium* isolates carrying pMAH135 genes displayed a specific VNTR genotype. Further work should be done to examine the characteristics of *M*. *avium* strains carrying pMAH135, especially in relation to the pathogenicity of these bacteria.

## Supporting Information

S1 Fig Amino acid sequence alignment of MAH_p85 and the multidrug and toxin extrusion (MATE) transporter NorM. Protein BLAST analysis revealed that MAH_p85 is a MATE family protein with putative conserved domains of the MATE transporter NorM from *Vibrio cholerae*. Asterisks indicate identical residues and dots indicate conservative amino acid substitutions. Rectangles represent residues constituting the cation-binding site and dashes represent gaps inserted to optimize the protein alignment. The alignment was performed by Clustal W at http://clustalw.ddbj.nig.ac.jp/.
(TIF)

S1 Table Primers used for detection of pMAH135 genes in *M*. *avium* isolates. (DOC)

S2 Table Summary of the 164 predicted coding sequences in pMAH135. (DOC)
