# Supplementary figures and images for: Characterization of a Novel Plasmid, pMAH135, from Mycobacterium avium Subsp. hominissuis
Source: PLoS One. 2015 Feb 11;10(2):e0117797. doi: 10.1371/journal.pone.0117797 (PMC4324632; doi:10.1371/journal.pone.0117797)

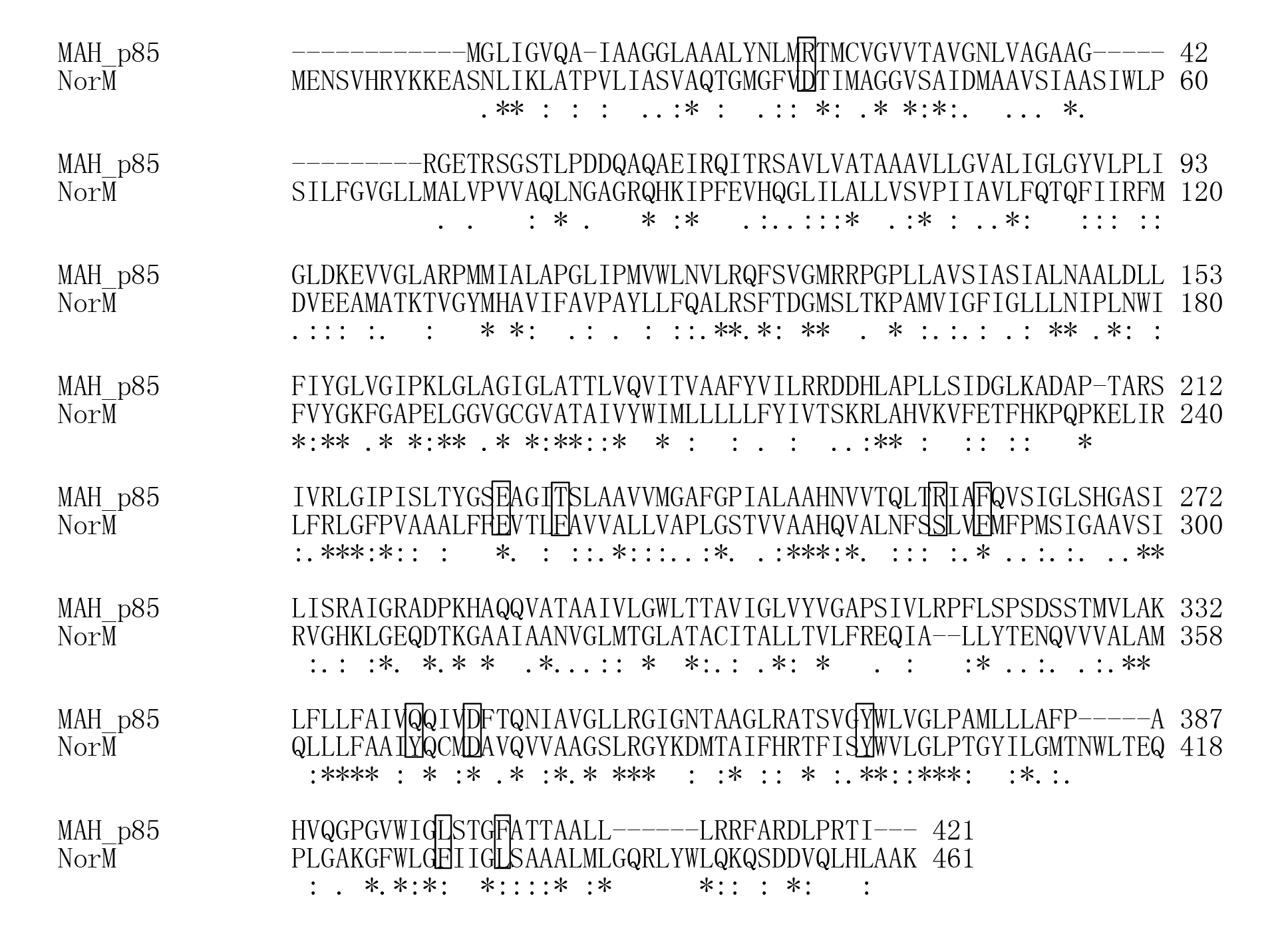

Supplement: S1 Fig — Protein BLAST analysis revealed that MAH_p85 is a MATE family protein with putative conserved domains of the MATE transporter NorM from Vibrio cholerae. Asterisks indicate identical residues and dots indicate conservative amino acid substitutions. Rectangles represent residues constituting the cation-binding site and dashes represent gaps inserted to optimize the protein alignment. The alignment was performed by Clustal W at http://clustalw.ddbj.nig.ac.jp/. (TIF) [file pone.0117797.s001.tif]
