# Supplementary material for: Characterization of a Novel Plasmid, pMAH135, from Mycobacterium avium Subsp. hominissuis
Source: PLoS One. 2015 Feb 11;10(2):e0117797. doi: 10.1371/journal.pone.0117797 (PMC4324632; doi:10.1371/journal.pone.0117797)
Supplement: S1 Table — (DOC) [file pone.0117797.s002.doc]

Table S1. Primers used for detection of pMAH135 genes in *M. avium* isolates

| Target gene | Product size (bp) | Nucleotide sequence |
| --- | --- | --- |
| MAH_p47  MAH_p49  MAH_p59  MAH_p85  MAH_p143  MAH_p148 | 457  1024  874  935  824  257 | F: 5’-GAACGCTCTCGGATACTTCG-3’  R: 5’-GAACAGGTCCAGGTCGGTTA-3’  F: 5’-ATGTACGAGAGTGGGGGACA-3’  R: 5’-CGTAGACCGACAGGTCGAAT-3’  F: 5’-CCTGGAAATAGCTTGGGACA-3’  R: 5’-GACAAGATCGACGGCAGATT-3’  F: 5’-GATGCGTACCATGTGCGTAG-3’  R: 5’-GCTTGGCCAACACCATAGTT-3’  F: 5’-TGGCGACATCCAGTAGGTCT-3’  R: 5’-TGAAGTCAACCTTTGCGTTG-3’  F: 5’-CTGTCGGTGCTGTTCATGTTGG-3’  R: 5’-TCGATCAATTACCAGTTCGGTG-3’ |
|  |  |  |
