# Supplementary material for: Characterization of a Novel Plasmid, pMAH135, from Mycobacterium avium Subsp. hominissuis
Source: PLoS One. 2015 Feb 11;10(2):e0117797. doi: 10.1371/journal.pone.0117797 (PMC4324632; doi:10.1371/journal.pone.0117797)
Supplement: S2 Table — (DOC) [file pone.0117797.s003.doc]

| Table S2. Summary of the 164 predicted coding sequences in pMAH135 | | | | | | |  |  |  |
| --- | --- | --- | --- | --- | --- | --- | --- | --- | --- |
| Feature | Start | End | Strand | Predicted  protein size (amino acids) | Amino acid identity in  overlap (%) | Size of  overlap (amino acids) | E value | Predicted product | Closest organism |
| MAH_p01 | 1 | 1218 | + | 405 | 78 | 405 | 0 | replication protein Rep | *Mycobacterium parascrofulaceum* |
| MAH_p02 | 1274 | 1882 | + | 202 | 50 | 171 | 4.00E-43 | Transcription factor WhiB | *M. smegmatis* |
| MAH_p03 | 1897 | 2274 | - | 125 | 58 | 125 | 2.00E-31 | conserved hypothetical protein | *M. parascrofulaceum* |
| MAH_p04 | 2267 | 3061 | - | 264 | 92 | 264 | 3.00E-176 | chromosome partitioning protein ParA | *M. parascrofulaceum* |
| MAH_p05 | 5068 | 5781 | + | 237 | 90 | 237 | 1.00E-154 | plasmid partitioning protein ParA | *M. tusciae* |
| MAH_p06 | 5781 | 6623 | + | 280 | 78 | 277 | 5.00E-153 | plasmid partitioning protein ParB | *M. parascrofulaceum* |
| MAH_p07 | 6839 | 7090 | + | 83 | 86 | 83 | 5.00E-43 | plasmid stability protein | *M. parascrofulaceum* |
| MAH_p08 | 7087 | 7503 | + | 138 | 86 | 137 | 5.00E-71 | PilT domain-containing protein | *M. parascrofulaceum* |
| MAH_p09 | 7608 | 8018 | - | 136 | 85 | 136 | 2.00E-72 | hypothetical protein | *M. tusciae* |
| MAH_p10 | 8224 | 8385 | + | 53 | 83 | 53 | 2.00E-22 | conserved hypothetical protein | *M. parascrofulaceum* |
| MAH_p11 | 8674 | 8859 | + | 61 | 35 | 48 | 4.1 | hypothetical protein Amet_3900 | *Alkaliphilus metalliredigens* QYMF |
| MAH_p12 | 9507 | 10658 | - | 383 | 93 | 383 | 0 | hypothetical protein Mkms_5598 | *M*. sp. KMS |
| MAH_p13 | 10747 | 11088 | + | 113 | 96 | 113 | 3.00E-72 | putative transcriptional regulatory protein | *M*. sp. KMS |
| MAH_p14 | 11085 | 11645 | + | 186 | 90 | 185 | 4.00E-118 | RES domain protein | *M. tusciae* |
| MAH_p15 | 11861 | 12211 | - | 116 | 76 | 112 | 3.00E-46 | conserved hypothetical protein | *M. parascrofulaceum* |
| MAH_p16 | 12208 | 13785 | - | 525 | 73 | 525 | 0 | conserved hypothetical protein | *M. parascrofulaceum* |
| MAH_p17 | 13688 | 14029 | + | 113 | 37 | 49 | 4.4 | putative permease | *Caulobacter* sp. AP07 |
| MAH_p18 | 13928 | 14233 | - | 101 | 82 | 101 | 2.00E-54 | conserved hypothetical protein | *M. parascrofulaceum* |
| MAH_p19 | 14309 | 14641 | - | 110 | 76 | 110 | 3.00E-52 | conserved hypothetical protein | *M. parascrofulaceum* |
| MAH_p20 | 14644 | 16272 | - | 542 | 76 | 475 | 8.00E-172 | conserved hypothetical protein | *M. parascrofulaceum* |
| MAH_p21 | 16363 | 17247 | - | 294 | 89 | 123 | 4.00E-65 | conserved hypothetical protein | *M. parascrofulaceum* |
| MAH_p22 | 17794 | 18993 | - | 399 | 61 | 324 | 6.00E-74 | conserved hypothetical protein | *M. parascrofulaceum* |
| MAH_p23 | 19223 | 19612 | - | 129 | 79 | 127 | 7.00E-63 | PilT domain-containing protein | *M. vanbaalenii* |
| MAH_p24 | 19609 | 19848 | - | 79 | 67 | 71 | 7.00E-25 | prevent-host-death family protein | *M. vaccae* |
| MAH_p25 | 19973 | 20902 | - | 309 | 86 | 309 | 0 | site-specific recombinase XerD | *M. chubuense* |
| MAH_p26 | 21189 | 22604 | - | 471 | 73 | 468 | 0 | NADH dehydrogenase (quinone) | *M. parascrofulaceum* |
| MAH_p27 | 22608 | 24098 | - | 496 | 85 | 496 | 0 | proton-translocating NADH-quinone oxidoreductase, chain M | *M*. sp. H4Y |
| MAH_p28 | 24102 | 25949 | - | 615 | 72 | 606 | 0 | NADH-ubiquinone oxidoreductase chain 5 | *M. indicus pranii* |
| MAH_p29 | 25946 | 26248 | - | 100 | 91 | 100 | 4.00E-55 | NADH-ubiquinone oxidoreductase | *M. parascrofulaceum* |
| MAH_p30 | 26245 | 26808 | - | 187 | 87 | 187 | 8.00E-93 | hypothetical protein W7S_14295 | *M*. sp. MOTT36Y |
| MAH_p31 | 26812 | 27747 | - | 311 | 76 | 308 | 7.00E-150 | NADH-quinone oxidoreductase subunit H 1 | *M. indicus pranii* |
| MAH_p32 | 27740 | 28825 | - | 361 | 54 | 361 | 2.00E-117 | hypothetical protein W7U_09865 | *M*. sp. H4Y |
| MAH_p33 | 28987 | 29331 | - | 114 | 79 | 114 | 1.00E-55 | NADH-ubiquinone/plastoquinone oxidoreductase chain 3 | *M*. sp. MOTT36Y |
| MAH_p34 | 29623 | 30009 | + | 128 | 87 | 113 | 4.00E-62 | conserved hypothetical protein | *M. parascrofulaceum* |
| MAH_p35 | 30180 | 31385 | - | 401 | 100 | 401 | 0 | ISMav6 protein | *M. avium* subsp. *hominissuis* |
| MAH_p36 | 31616 | 32041 | + | 141 | 91 | 141 | 2.00E-88 | conserved hypothetical protein | *M. parascrofulaceum* |
| MAH_p37 | 32481 | 32687 | + | 68 | 68 | 68 | 1.00E-24 | heavy metal transport/detoxification protein | *M. parascrofulaceum* |
| MAH_p38 | 32726 | 34975 | + | 749 | 79 | 740 | 0 | ctpB cation transporter, P-type ATPase B | *Nocardia cyriacigeorgica* |
| MAH_p39 | 35179 | 35583 | - | 134 | 91 | 131 | 3.00E-71 | MerR family transcriptional regulator | *M. intracellulare* |
| MAH_p40 | 36174 | 37040 | - | 288 | 83 | 207 | 1.00E-101 | hypothetical protein OCU_23180 | *M. intracellulare* |
| MAH_p41 | 37668 | 38606 | - | 312 | 67 | 275 | 4.00E-121 | putative hydrolase (alpha/beta hydrolase fold) | *M. abscessus* |
| MAH_p42 | 38629 | 38928 | + | 99 | 29 | 82 | 0.75 | peptidyl-tRNA hydrolase | *Dietzia alimentaria* |
| MAH_p43 | 39022 | 42021 | - | 999 | 60 | 998 | 0 | polyketide synthase MbtD | *M. indicus pranii* |
| MAH_p44 | 42018 | 43307 | - | 429 | 81 | 424 | 0 | polyketide synthase MbtC | *M. indicus pranii* |
| MAH_p45 | 43304 | 44038 | - | 244 | 70 | 243 | 5.00E-116 | thioesterase | *M*. sp. MOTT36Y |
| MAH_p46 | 44035 | 44613 | - | 192 | 64 | 185 | 2.00E-75 | Rhizobactin siderophore biosynthesis protein rhbD | *M. indicus pranii* |
| MAH_p47 | 44693 | 49681 | + | 1662 | 67 | 1662 | 0 | non-ribosomal peptide synthetase MbtE | *M*. sp. MOTT36Y |
| MAH_p48 | 49678 | 54141 | + | 1487 | 69 | 1476 | 0 | non-ribosomal peptide synthetase MbtF | *M*. sp. MOTT36Y |
| MAH_p49 | 54165 | 57617 | + | 1150 | 72 | 1147 | 0 | phenyloxazoline synthase MbtB | *M. marinum* |
| MAH_p50 | 57618 | 58982 | - | 454 | 71 | 424 | 0 | hypothetical protein W7S_14320 | *M*. sp. MOTT36Y |
| MAH_p51 | 59447 | 59731 | - | 94 | 91 | 94 | 2.00E-56 | hypothetical protein MintA_21669 | *M. intracellulare* |
| MAH_p52 | 59742 | 60038 | - | 98 | 92 | 98 | 2.00E-60 | EsaT-6 like protein EsxP | *M. parascrofulaceum* |
| MAH_p53 | 60132 | 61337 | - | 401 | 72 | 401 | 2.00E-132 | PPE family protein | *M*. sp. MOTT36Y |
| MAH_p54 | 61351 | 61650 | - | 99 | 72 | 87 | 7.00E-34 | PE family protein | *M. marinum* |
| MAH_p55 | 62761 | 66741 | - | 1326 | 74 | 1283 | 0 | amino acid adenylation enzyme/thioester reductase family protein | *M. rhodesiae* |
| MAH_p56 | 66738 | 67979 | - | 413 | 76 | 349 | 0 | pyridoxal-5'-phosphate-dependent enzyme subunit beta | *M*. sp. JLS |
| MAH_p57 | 68022 | 68402 | - | 126 | 55 | 126 | 6.00E-24 | LpqS family protein, partial | *M. parascrofulaceum* |
| MAH_p58 | 68931 | 84137 | - | 5068 | 59 | 5068 | 0 | syringomycin synthetase | *M. intracellulare* |
| MAH_p59 | 84176 | 85720 | - | 514 | 56 | 491 | 0 | drug transporter | *M. parascrofulaceum* |
| MAH_p60 | 85747 | 86691 | - | 314 | 81 | 312 | 0 | formyl transferase domain protein | *M. tusciae* |
| MAH_p61 | 87325 | 87477 | + | 50 |  |  |  |  |  |
| MAH_p62 | 87458 | 87640 | - | 60 | 37 | 43 | 2.6 | succinate--CoA ligase | *Thermocrinis albus* |
| MAH_p63 | 87651 | 88298 | - | 215 | 60 | 193 | 3.00E-69 | hypothetical protein W7S_14365 | *M*. sp. MOTT36Y |
| MAH_p64 | 88368 | 88628 | - | 86 | 45 | 45 | 1.00E-04 | hypothetical protein A20C1_00010 | marine actinobacterium PHSC20C1 |
| MAH_p65 | 88967 | 89305 | + | 112 | 70 | 111 | 5.00E-44 | hypothetical protein W7U_25365 | *M*. sp. H4Y |
| MAH_p66 | 90010 | 91263 | + | 417 | 88 | 417 | 0 | putative lipoprotein | *M. avium* |
| MAH_p67 | 91572 | 92432 | - | 286 | 87 | 286 | 0 | ISMsm7 transposase | *M. parascrofulaceum* |
| MAH_p68 | 92429 | 92737 | - | 102 | 85 | 102 | 1.00E-56 | transposase IS3/IS911 | *M. smegmatis* |
| MAH_p69 | 92853 | 93134 | + | 93 | 44 | 40 | 1.6 | LysR substrate binding domain protein | *Neisseria* sp. oral taxon 020 |
| MAH_p70 | 93024 | 94394 | - | 456 | 79 | 455 | 0 | PPE family protein | *M. intracellulare* |
| MAH_p71 | 94796 | 95563 | + | 255 | 66 | 228 | 1.00E-86 | hypothetical protein MMAR_2870 | *M. marinum* |
| MAH_p72 | 96562 | 96852 | - | 96 | 84 | 96 | 5.00E-52 | hypothetical protein MMAR_1447 | *M. marinum* |
| MAH_p73 | 97171 | 99588 | - | 805 | 83 | 792 | 0 | P-ATPase superfamily P-type ATPase copper transporter | *M. parascrofulaceum* |
| MAH_p74 | 99578 | 99874 | - | 98 | 69 | 97 | 5.00E-35 | hypothetical protein Rv0968 | *M. tuberculosis* H37Rv |
| MAH_p75 | 99957 | 100316 | - | 119 | 87 | 118 | 1.00E-66 | copper-sensing transcriptional repressor CsoR | *M. parascrofulaceum* |
| MAH_p76 | 100433 | 101188 | + | 251 | 66 | 251 | 1.00E-87 | conserved hypothetical protein | *M. parascrofulaceum* |
| MAH_p77 | 101858 | 103315 | - | 485 | 72 | 477 | 0 | Copper resistance protein A -like protein | *M. indicus pranii* |
| MAH_p78 | 104078 | 104398 | - | 106 | 74 | 104 | 7.00E-48 | hypothetical protein Mspyr1_54620 | *M. gilvum* Spyr1 |
| MAH_p79 | 104654 | 104995 | - | 113 | 46 | 37 | 0.46 | conserved domain protein | *Delta proteobacterium* NaphS2 |
| MAH_p80 | 104885 | 105202 | + | 105 | 53 | 98 | 2.00E-21 | lipoprotein LpqS | *M. ulcerans* |
| MAH_p81 | 105451 | 106551 | + | 366 | 79 | 339 | 0 | cysteine synthase a CysK2 | *M. marinum* |
| MAH_p82 | 106592 | 106897 | + | 101 | 67 | 69 | 1.00E-21 | hypothetical protein W7U_09690 | *M*. sp. H4Y |
| MAH_p83 | 107156 | 108175 | + | 339 | 83 | 339 | 0 | ornithine cyclodeaminase | *M. indicus pranii* |
| MAH_p84 | 108166 | 109536 | + | 456 | 76 | 456 | 0 | diaminopimelate decarboxylase | *M. indicus pranii* |
| MAH_p85 | 109677 | 110942 | + | 421 | 80 | 421 | 0 | MATE efflux family protein | M. sp. MOTT36Y |
| MAH_p86 | 111406 | 112212 | + | 268 |  |  |  |  |  |
| MAH_p87 | 113235 | 113633 | - | 132 | 37 | 126 | 2.00E-07 | hypothetical protein W7U_10090 | *M*. sp. H4Y |
| MAH_p88 | 113694 | 115082 | - | 462 | 72 | 411 | 0 | major facilitator superfamily MFS_1 | *M. smegmatis* |
| MAH_p89 | 115079 | 116320 | - | 413 | 76 | 349 | 0 | pyridoxal-5'-phosphate-dependent enzyme subunit beta | *M*. sp. JLS |
| MAH_p90 | 116363 | 116743 | - | 126 | 55 | 126 | 6.00E-24 | LpqS family protein, partial | *M. parascrofulaceum* |
| MAH_p91 | 117308 | 118867 | + | 519 | 52 | 501 | 7.00E-127 | PPE family protein | *M. marinum* |
| MAH_p92 | 120031 | 120264 | + | 77 | 83 | 77 | 3.00E-34 | hypothetical protein Mycch_2963 | *M. chubuense* |
| MAH_p93 | 120254 | 120565 | + | 103 | 84 | 103 | 2.00E-55 | growth inhibitor | *M. smegmatis* |
| MAH_p94 | 120716 | 121132 | - | 138 | 79 | 133 | 6.00E-73 | arsenate reductase | *M. parascrofulaceum* |
| MAH_p95 | 121129 | 122232 | - | 367 | 84 | 354 | 0 | arsenic-transport integral membrane protein ArsC | *M. abscessus* |
| MAH_p96 | 122267 | 122746 | - | 159 | 77 | 159 | 3.00E-81 | Glyoxalase/bleomycin resistance protein/dioxygenase | *M. rhodesiae* |
| MAH_p97 | 122849 | 123208 | + | 119 | 85 | 118 | 9.00E-67 | ArsR family transcriptional regulator | *M. vaccae* |
| MAH_p98 | 124265 | 124819 | + | 184 | 37 | 129 | 2.00E-10 | hypothetical protein | *Thermaerobacter marianensis* |
| MAH_p99 | 124985 | 125266 | + | 93 | 96 | 93 | 1.00E-56 | transposase, ISMyma01_aa1 | *M*. sp. H4Y |
| MAH_p100 | 125308 | 126102 | + | 264 | 91 | 264 | 6.00E-174 | ISDet2 transposase | *M. parascrofulaceum* |
| MAH_p101 | 126334 | 127056 | - | 240 | 38 | 209 | 1.00E-31 | GCN5-related N-acetyltransferase | *M. tusciae* |
| MAH_p102 | 127755 | 129401 | + | 548 | 89 | 548 | 0 | nuclease | *M. parascrofulaceum* |
| MAH_p103 | 130125 | 130358 | + | 77 | 90 | 77 | 2.00E-44 | glutaredoxin | *M. parascrofulaceum* |
| MAH_p104 | 130386 | 130679 | + | 97 | 79 | 97 | 3.00E-47 | conserved hypothetical protein | *M. parascrofulaceum* |
| MAH_p105 | 130679 | 131965 | + | 428 | 73 | 428 | 0 | conserved hypothetical protein | *M. parascrofulaceum* |
| MAH_p106 | 131962 | 132132 | + | 56 | 96 | 56 | 2.00E-17 | hypothetical protein HMPREF0591_1392 | *M. parascrofulaceum* |
| MAH_p107 | 132157 | 132882 | + | 241 | 80 | 240 | 3.00E-132 | conserved hypothetical protein | *M. parascrofulaceum* |
| MAH_p108 | 133404 | 133940 | + | 178 | 75 | 175 | 2.00E-92 | conserved hypothetical protein | *M. parascrofulaceum* |
| MAH_p109 | 134351 | 134701 | + | 116 | 69 | 116 | 3.00E-46 | conserved hypothetical protein | *M. parascrofulaceum* |
| MAH_p110 | 134821 | 135804 | + | 327 | 82 | 327 | 2.00E-170 | conserved hypothetical protein | *M. parascrofulaceum* |
| MAH_p111 | 136220 | 136681 | + | 153 | 88 | 145 | 6.00E-88 | conserved hypothetical protein, partial | *M. parascrofulaceum* |
| MAH_p112 | 136765 | 137100 | + | 111 | 58 | 110 | 3.00E-31 | hypothetical protein MycrhDRAFT_3756 | *M. rhodesiae* |
| MAH_p113 | 137503 | 137961 | + | 152 | 86 | 109 | 8.00E-65 | conserved hypothetical protein, partial | *M. parascrofulaceum* |
| MAH_p114 | 137989 | 138165 | + | 58 | 68 | 56 | 4.00E-19 | cobalamin biosynthesis protein CobP | *M. parascrofulaceum* |
| MAH_p115 | 138575 | 139525 | + | 316 | 78 | 314 | 3.00E-151 | conserved hypothetical protein | *M. parascrofulaceum* |
| MAH_p116 | 140000 | 141046 | + | 348 | 91 | 348 | 0 | conserved hypothetical protein | *M. parascrofulaceum* |
| MAH_p117 | 141395 | 141919 | - | 174 | 84 | 174 | 2.00E-91 | conserved hypothetical protein | *M. parascrofulaceum* |
| MAH_p118 | 142007 | 142501 | - | 164 | 72 | 153 | 2.00E-40 | hypothetical protein HMPREF0591_1280 | *M. parascrofulaceum* |
| MAH_p119 | 142789 | 148620 | + | 1943 | 77 | 1943 | 0 | exonuclease V subunit alpha | *M. parascrofulaceum* |
| MAH_p120 | 148617 | 149888 | - | 423 | 85 | 394 | 0 | transposase IS891/IS1136/IS1341 family | *M. tusciae* |
| MAH_p121 | 149996 | 150256 | + | 86 | 69 | 60 | 9.00E-18 | conserved hypothetical protein | *M. parascrofulaceum* |
| MAH_p122 | 150289 | 151746 | - | 485 | 56 | 485 | 2.00E-98 | conserved hypothetical protein | *M. parascrofulaceum* |
| MAH_p123 | 151743 | 152348 | - | 201 | 53 | 167 | 2.00E-42 | hypothetical protein MULP_087 | *M. liflandii* |
| MAH_p124 | 152787 | 153560 | + | 257 | 36 | 234 | 1.00E-37 | hypothetical protein SCAT_5663 | *Streptomyces cattleya* |
| MAH_pt01 | 153593 | 153668 | - |  |  |  |  |  |  |
| MAH_p125 | 153774 | 154097 | - | 107 | 51 | 76 | 2.00E-15 | gp82 | *M*. phage Barnyard |
| MAH_p126 | 154109 | 154258 | - | 49 | 76 | 49 | 9.00E-19 | hypothetical protein HMPREF0591_1290 | *M. parascrofulaceum* |
| MAH_p127 | 154251 | 154685 | - | 144 | 44 | 124 | 9.00E-19 | conserved hypothetical protein | *M. parascrofulaceum* |
| MAH_p128 | 154682 | 154966 | - | 94 | 68 | 77 | 4.00E-23 | conserved hypothetical protein | *M. parascrofulaceum* |
| MAH_p129 | 154963 | 156378 | - | 471 | 82 | 464 | 0 | type IV secretory pathway VirD4 family protein | *M. parascrofulaceum* |
| MAH_p130 | 156381 | 156836 | - | 151 | 84 | 150 | 6.00E-72 | conserved hypothetical protein | *M. parascrofulaceum* |
| MAH_p131 | 157030 | 157464 | - | 144 | 85 | 144 | 5.00E-72 | conserved hypothetical protein | *M. parascrofulaceum* |
| MAH_p132 | 157564 | 157854 | + | 96 | 46 | 96 | 6.00E-13 | conserved hypothetical protein | *M. parascrofulaceum* |
| MAH_p133 | 157851 | 159107 | + | 418 | 70 | 342 | 1.00E-147 | conserved hypothetical protein | *M. parascrofulaceum* |
| MAH_p134 | 159125 | 160048 | + | 307 | 92 | 304 | 0 | conserved hypothetical protein | *M. parascrofulaceum* |
| MAH_p135 | 160058 | 160258 | + | 66 | 45 | 47 | 2.2 | hypothetical protein Spro_0898 | *Serratia proteamaculans* |
| MAH_p136 | 160314 | 161519 | - | 401 | 100 | 401 | 0 | ISMav6 protein | *M. avium* subsp. *Hominissuis* |
| MAH_p137 | 161750 | 162034 | + | 94 | 84 | 94 | 1.00E-44 | family 39 glycosyl transferase | *M. parascrofulaceum* |
| MAH_p138 | 162108 | 165089 | + | 993 | 88 | 993 | 0 | conserved hypothetical protein | *M. parascrofulaceum* |
| MAH_p139 | 165086 | 167161 | + | 691 | 76 | 690 | 0 | conserved hypothetical protein | *M. parascrofulaceum* |
| MAH_p140 | 167158 | 167622 | + | 154 | 61 | 145 | 3.00E-47 | hypothetical protein MA5S1215_2120 | *M. abscessus* |
| MAH_p141 | 167629 | 168072 | - | 147 | 46 | 121 | 3.00E-18 | hypothetical protein MA6G0125S_5419 | *M. abscessus* |
| MAH_p142 | 168079 | 169917 | - | 612 | 91 | 610 | 0 | type VII secretion AAA-ATPase EccA | *M. parascrofulaceum* |
| MAH_p143 | 169933 | 171528 | - | 531 | 79 | 531 | 0 | type VII secretion protein EccE | *M. parascrofulaceum* |
| MAH_p144 | 171525 | 173237 | - | 570 | 88 | 515 | 0 | type VII secretion-associated serine protease mycosin | *M. parascrofulaceum* |
| MAH_p145 | 173252 | 174766 | - | 504 | 90 | 504 | 0 | ｔype VII secretion integral membrane protein EccD | *M. parascrofulaceum* |
| MAH_p146 | 174763 | 176271 | - | 502 | 78 | 291 | 5.00E-159 | conserved hypothetical protein | *M. parascrofulaceum* |
| MAH_p147 | 176396 | 177316 | - | 306 | 84 | 305 | 6.00E-171 | conserved hypothetical protein | *M. parascrofulaceum* |
| MAH_p148 | 177399 | 177683 | - | 94 | 98 | 94 | 2.00E-59 | EsaT-6 like protein EsxN | *M. parascrofulaceum* |
| MAH_p149 | 177727 | 178023 | - | 98 | 98 | 98 | 2.00E-64 | EsaT-6 like protein EsxP | *M. parascrofulaceum* |
| MAH_p150 | 178166 | 179329 | - | 387 | 87 | 387 | 0 | PPE family protein | *M. parascrofulaceum* |
| MAH_p151 | 179360 | 179656 | - | 98 | 90 | 98 | 1.00E-38 | PE family protein | *M. parascrofulaceum* |
| MAH_p152 | 179751 | 183965 | - | 1404 | 81 | 1404 | 0 | type VII secretion protein EccCa | *M. parascrofulaceum* |
| MAH_p153 | 183962 | 185482 | - | 506 | 91 | 506 | 0 | type VII secretion protein EccB | *M. parascrofulaceum* |
| MAH_p154 | 185565 | 186329 | - | 254 | 88 | 112 | 9.00E-66 | NLP/P60 family protein | *M. parascrofulaceum* |
| MAH_p155 | 186348 | 187289 | - | 313 | 73 | 279 | 6.00E-141 | hypothetical protein HMPREF0591_1322 | *M. parascrofulaceum* |
| MAH_p156 | 187380 | 187724 | - | 114 | 48 | 108 | 6.00E-26 | GCN5-related N-acetyltransferase | *Microbacterium laevaniformans* |
| MAH_p157 | 187830 | 189938 | - | 702 | 72 | 699 | 0 | Possible methyl-accepting chemotaxis sensory | *M. parascrofulaceum* |
| MAH_p158 | 190170 | 190655 | - | 161 | 48 | 121 | 3.00E-32 | hypothetical protein MycrhDRAFT_6958 | *M. rhodesiae* |
| MAH_p159 | 190847 | 191116 | - | 89 | 97 | 89 | 7.00E-35 | oxidoreductase | *M. kansasii* |
| MAH_p160 | 191310 | 192005 | - | 231 | 90 | 230 | 1.00E-143 | hypothetical protein | *M. kansasii* |
| MAH_p161 | 192037 | 193089 | - | 350 | 64 | 341 | 5.00E-140 | conserved hypothetical protein | *M. parascrofulaceum* |
| MAH_p162 | 193086 | 193274 | - | 62 | 80 | 61 | 5.00E-23 | XRE family transcriptional regulator | *M. parascrofulaceum* |
| MAH_p163 | 193499 | 193795 | + | 98 | 75 | 98 | 7.00E-46 | transcription factor WhiB family protein | *M. parascrofulaceum* |
| MAH_p164 | 193805 | 194089 | + | 94 | 76 | 90 | 1.00E-40 | conserved hypothetical protein | *M. parascrofulaceum* |
